# Calsyntenin-1, clusterin and neutrophil gelatinase-associated lipocalin are candidate serological biomarkers for lung adenocarcinoma

**DOI:** 10.18632/oncotarget.22438

**Published:** 2017-11-14

**Authors:** Yen Chu, Yu-Heng Lai, Ming-Cheng Lee, Yu-Jung Yeh, Yao-Kuang Wu, Wayne Tsao, Chun-Yao Huang, Semon Wu

**Affiliations:** ^1^ Department of Medical Research and Development, Chang Gung Memorial Hospital, Graduate Institute of Traditional Chinese Medicine, Chang Gung University, Taoyuan, Taiwan; ^2^ Department of Chemistry, Chinese Culture University, Taipei, Taiwan; ^3^ Department of Research, Taipei Tzu Chi Hospital, The Buddhist Tzu Chi Medical Foundation, Taipei, Taiwan; ^4^ Department of Mathematics & Statistics, San Jose State University, San Jose, California, USA; ^5^ Department of Pulmonary and Critical Care, Taipei Tzu Chi Hospital, The Buddhist Tzu Chi Medical Foundation, Taipei, Taiwan; ^6^ Department of Life Science, Chinese Culture University, Taipei, Taiwan

**Keywords:** serological biomarkers, non-small cell lung cancer, proteomic

## Abstract

It has been drawn attention that secreted proteins with signal peptide from cancer cells provide new potential biomarkers of cancer. In this study, three lung adenocarcinoma cell lines and serum samples from 20 patients were used for identifying potential serologic tumor biomarker with proteomic and bioinformatics approaches. One-dimensional electrophoresis, and identified with mass spectrometry and database research were performed. We found17 secreted proteins in common, while another 17 proteins with signal peptide were identified in all three lung adenocarcinoma cell lines alone with patient samples. With matching these two groups of identified proteins, calsyntenin-1 (CLSTN1), clusterin (CLU) and neutrophil gelatinase-associated lipocalin (NGAL) were found highly secreted from both cell lines and serum with unique signal peptides. Therefore, in our study, we demonstrated that cancer cells secret specific proteins to the environment that may serve as unique markers for cancer diagnosis. To combination of proteomic study with bioinformatic prediction on signal peptides, higher expression level of CLSTN1, CLU and NGAL were found and may be new solid serologic biomarkers for patients with lung adenocarcinoma.

## INTRODUCTION

Lung cancer is one of the most common cancers which shows leading cause of cancer mortality in many countries, including Taiwan. Upon cancer biological research, serological biomarker screening plays a key role in monitoring of cancer progression, treatment response and surveillance for recurrence. It is known that proteins secreted from cancer cells can flow into bloodstream. However, when secreted proteins are over-expressed, they may induce the occurrence of carcinogenesis, which show potential to be serological biomarkers of cancer. Previous studies have identified numerous serological biomarkers on different malignant tumors, such as macrophage inhibitory cytokine-1 in metastatic prostate, breast, and colorectal carcinomas, osteopontin and prostasin in ovarian carcinomas [1–3].

However, serum protein biomarkers of lung cancer, such as neuron-specific enolase, carcinoembryonic antigen (CEA), carbohydrate antigen (CA) 125, cytokeratin 19 fragment (Cyfra21), and Dickkopf-1 (Dkk1) may not be promising for detection lung cancer due to limited specificity and sensitivity and large number of false-positive findings [4–7]. Previously, our publication successfully demonstrated increased precision and accuracy on serological lung cancer biomarker through 2-D liquid phase fractionation system (PF2D) and mass spectrometry approach and identified haptoglobin as a solid candidate [8]. In addition, high serum haptoglobin level has been suggested as a key biomarker associated with tumor progression and prognosis in non-small cell lung cancer [9]. These evidences may confirm the power of serological markers on clinical application.

However, huge demand of manpower and time-consuming procedures may impede cancer research. Thus, the urgency and necessity to efficiently identify solid candidates of serological biomarkers for lung cancer diagnostics always has its priority.

A number of proteomic studies with serum free cell culture model were performed for searching novel cancer biomarkers [3, 7, 10, 11]. There are two types of secretion proteins released to cell-cultured medium. The first kind of proteins is exported via classical protein export pathway with signal peptide recognition. The other is secreted through exosome-mediated pathway. It has been well-known that circulating proteins with signal peptide could be biomarkers for cancer [12, 13]. In addition, more evidence demonstrated that exosomes involved in cancer progression [14, 15]. These suggested that serological biomarkers show great contribution on cancer screening and may draw more attention on predictive strategy on cancer diagnosis.

Therefore, three lung adenocarcinoma cell lines, including A549 and two primary cell lines that were established from lung adenocarcinoma solid tumor were used in our study. With combination of proteomics and bioinformatics predictions on signal peptides, we try to identify potential serological biomarkers on lung cancer.

## RESULTS

### Identification of secreted proteins from lung adenocarcinoma cell lines

**Figure 1:**
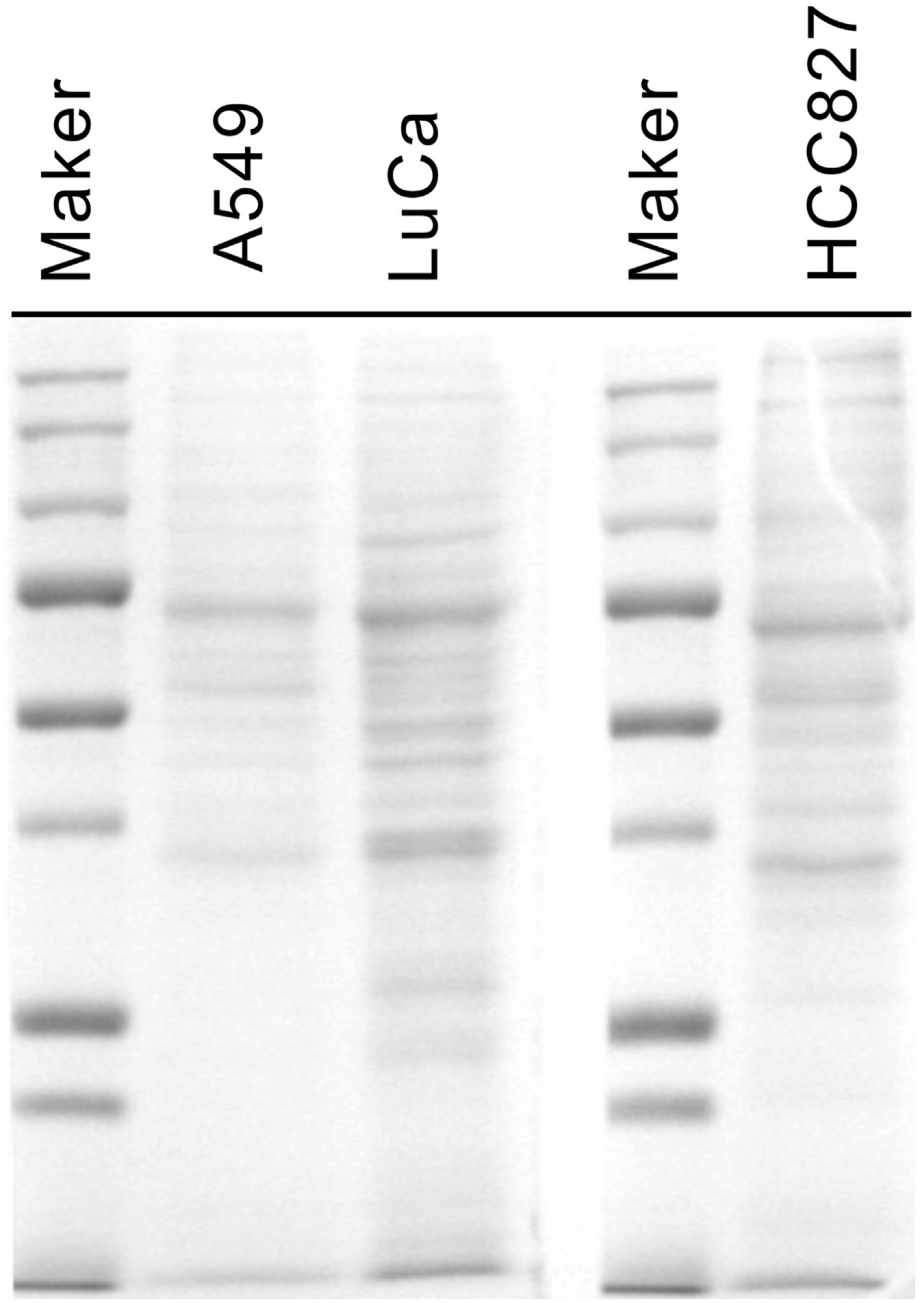
One-dimensional SDS PAGE of conditioned medium Lane 1: protein biomarkers; Lane 2: A549 cell line; Lane 3: Luca cell line; Lane 4: marker; Lane 5: HCC827 (Luca Chen) cell line.

To investigate potential biomarkers of lung cancer, we analyzed secreted proteins from three lung adenocarcinoma cell lines, including A549, LuCa and HCC827 cells. Cells were cultured in RPMI-1640 medium without serum that could avoid the albumin contamination from serum. Each culture supernatant was harvested, concentrated, and 30 μg total proteins of each cell lines was resolved on 10% SDS-PAGE and checked with Coomassie blue staining (Figure 1). Each lane of the gel was excised into 26 parts, independently in-gel digested with trypsin, and identified by MALDI-TOF MS. The identified proteins existed in triplicate on each cell line were included in this study. There are 32 proteins were identified from A549, 62 from LuCa and 49 from HCC827, respectively (Supplementary Tables 1-3). Totally, eighty-six different proteins were existed in this study.

### Allocation of the identified proteins into functional groups

**Figure 2:**
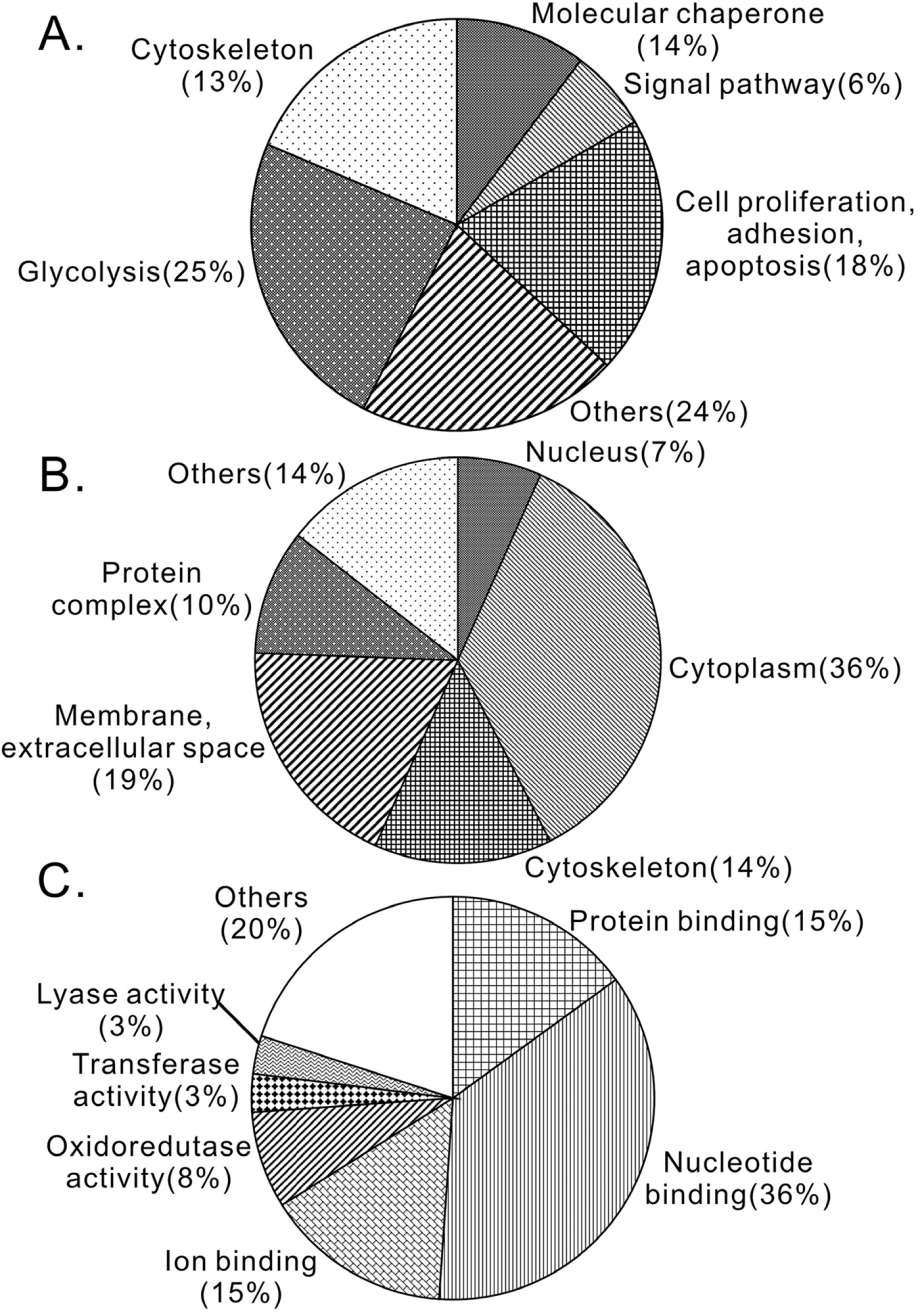
Identified proteins were classified into functional groups **(A)** Biological functional analysis. **(B)** Cellular component analysis. **(C)** Molecular functional analysis.

Based on Gene Ontology (GO) analysis, a total of 86 identified proteins from three-cancer cell lines were categorized into six subgroups according to biological functions, six subgroups according to different cellular components and seven subgroups according to molecular function (Figure 2). Among biological function grouping, 14% of identified proteins were involved in molecular chaperone; 6% in signal pathway; 18% in cell proliferation, adhesion and apoptosis; 25% in glycolysis; 13% in cytoskeleton and 24% were labeled “others” (Figure 2A). Among cellular component analysis, 7% of identified proteins were located on nucleus; 36% cytoplasm; 14% on cytoskeleton; 19% on extracellular space; 10% on protein complex and 14% were labeled “others” (Figure 2B). Among molecular function analysis, there are 15% of identified proteins were implicated in protein binding; 36% in nucleotide binding; 15% in ion binding; 8% in oxidoreductase activity; 3% in transporter activity; 3% in lyase activity and 20% were labeled “others” (Figure 2C).

Interestingly, there were 17 common proteins (14%), appearing in all three lung adenocarcinoma cell lines (Table 1), although large portion of heterogenesis was existed between these cell lines. Among these 17 commonly secreted proteins, there were 7 proteins involving in glycolysis, 5 proteins in cell proliferation, 2 proteins in chaperone, 2 proteins in cytoskeleton, 1 protein in transport and 1 protein in cell adhesion (Table 1).

**Table 1:** Secreted protein existed in all three NSCLC cell lines

Biological function	Gene ID	Protein Swiss number	Protein name	Mass (kDa)	PI
Glycolysis					
	7167	P60174	Triosephosphate isomerase	26.943	6.45
	2597	P04406	Glyceraldehyde-3-phosphate dehydrogenase	36.204	8.57
	226	P04075	Fructose-bisphosphate aldolase A	39.859	8.3
	5230	P00558	Phosphoglycerate kinase 1	44.992	8.3
	2023	P06733	Alpha-enolase	47.487	7.01
	216	P00352	Retinal dehydrogenase 1	55.465	6.3
	5315	P14618	Pyruvate kinase isozymes M1/M2	58.48	7.96
Cell proliferation					
	5052	Q06830	Peroxiredoxin-1	22.328	8.27
	2950	P09211	Glutathione S-transferase P	23.573	5.43
	57016	O60218	Aldo-keto reductase family 1 member B10	36.23	7.12
	1191	P10909	Clusterin precursor	53.041	5.89
Chaperone					
	3326	P08238	Heat shock protein HSP 90-beta	83.56	4.97
	3320	P07900	Heat shock protein HSP 90-alpha	85.013	4.94
Cytoskeleton					
	302	P07355	Annexin A2	38.812	7.57
	60	P60709	Actin, cytoplasmic 1	42.058	5.29
Transport					
	3934	P80188	Neutrophil gelatinase-associated lipocalin precursor	22.748	9.02
Cell adhesion					
	22883	O94985	Calsyntenin-1 precursor	111	4.96

### Prediction of secreted proteins by signalP3.0

To clarify whether 86 identified proteins were secreted through classical secretion pathway with signal peptides, amino acid sequences of the identified proteins were analyzed with SignalP3.0 program. We found that 17 unique proteins appearing in either one of cell lines, carried with the signal peptides for secretion (Table 2). Only 14% of identified proteins were predicted as classic secreted protein with signal peptide, indicating that majority of identified proteins released into the medium through a non-classic secretion pathway.

**Table 2:** The identified secreted proteins with signal peptide by SignalP3.0 signal peptide prediction server

Gene ID	Protein Swiss number	Protein name	Mass(kDa)	PI
3934	P80188	Neutrophil gelatinase-associated lipocalin precursor	22.748	9.02
4316	P09237	Matrilysin precursor	29.832	7.74
1514	P07711	Cathepsin L precursor	38.004	5.31
5328	P00749	Urokinase-type plasminogen activator precursor	49.944	8.78
1191	P10909	Clusterin precursor	53.041	5.89
2799	P15586	N-acetylglucosamine-6-sulfatase precursor	62.854	8.6
3959	Q08380	Galectin-3-binding protein precursor	66.217	5.13
3309	P11021	78 kDa glucose-regulated protein precursor	72.404	5.07
7045	Q15582	Transforming growth factor-beta-induced protein ig-h3 precursor	75.272	7.62
10131	Q12931	Heat shock protein 75 kDa, mitochondrial precursor	80.350	8.3
5768	O00391	Sulfhydryl oxidase 1 precursor	83.338	9.13
22883	O94985	Calsyntenin-1 precursor	111	4.81
718	P01024	Complement C3 precursor	188.596	6.02
375790	O00468	Agrin precursor	223.001	6.02
3371	P24821	Tenascin precursor	246.456	4.79
2335	P02751	Fibronectin precursor	266.096	5.45
3911	O15230	Laminin subunit alpha-5 precursor	412.258	6.61

### Validation of secreted proteins by western blotting

Taken together with the 17 common secreted proteins in three cell lines and the 17 unique proteins with predicted signal peptides, we found three proteins in common, which are CLSTN1, CLU and NGAL. On the basis of GO analysis, the biological function of these three proteins was involved in cell adhesion, proliferation and transport, respectively (Table 3).

**Table 3:** Gene ontology of calsyntenin-1, clusterin and neutrophil gelatinase-associated lipocalin by GeneCards^®^ server

Protein name	Cellular component	Molecular function	Biological process
Calsyntenin-1 precursor	plasma membrane, membrane, integral to membrane	calcium ion binding, protein binding	cell adhesion, homophilic cell adhesion
Clusterin precursor	perinuclear region of cytoplasm, aggresome, extracellular space	protein binding	apoptosis, anti-apoptosis, complement activation, response to oxidative stress, lipid metabolic process
Neutrophil gelatinase-associated lipocalin precursor	soluble fraction, cytoplasm	transporter activity, binding, pheromone binding	transport, lipid metabolic process

**Figure 3:**
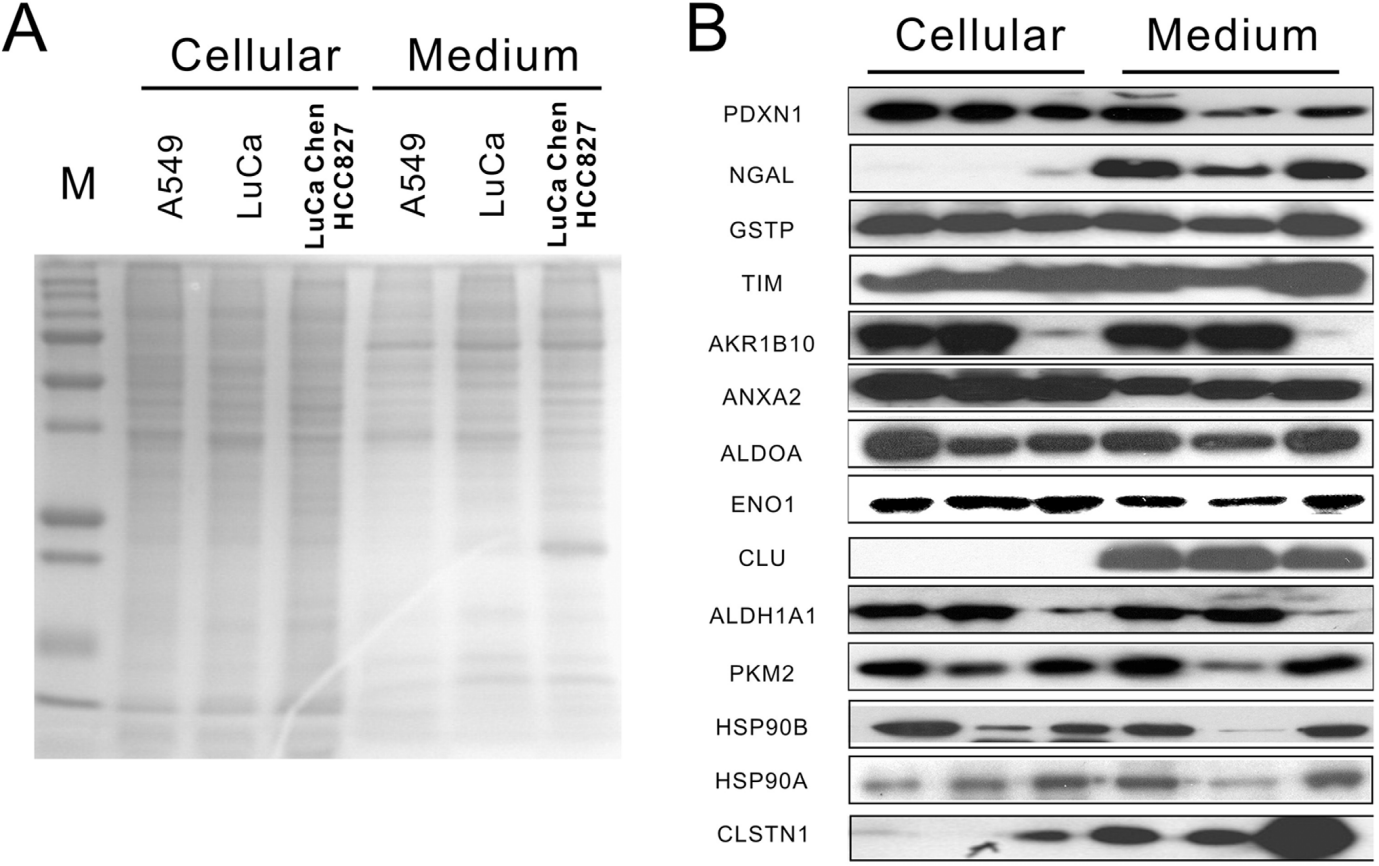
Protein expression of identified secreted proteins in cellular and conditioned medium **(A)** 1D SDS-PAGE of cellular and condition medium from three cell lines. **(B)** Western blotting results of identified proteins, including CLSTN1, CLU and NGAL in cellular environment and conditioned medium from three cell lines.

**Figure 4:**
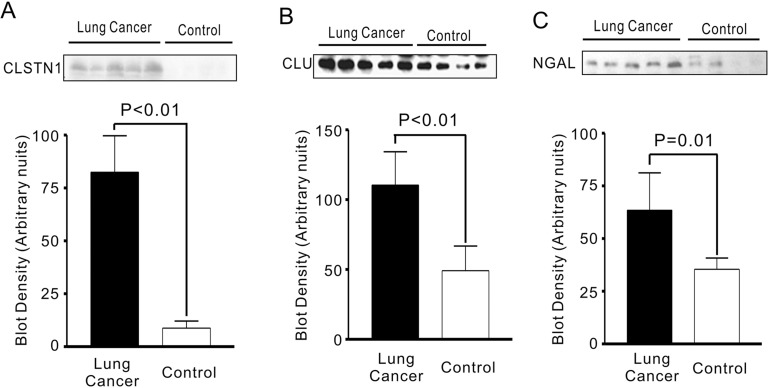
CLSTN1, CLU and NGAL expressions in serum from lung cancer patients and health controls **(A)** CLSTN1; **(B)** CLU; **(C)** NGAL.

CLSTN1, CLU, and NGAL were further validated by Western blotting for expression levels (Figure 3). Total cellular extract and condition medium from three lung cancer cell lines was separated with 12% SDS-polyacrylamide gel (Figure 3A). As shown in Figure 3B, increased expression levels of CLSTN1, CLU and NGAL were found in conditioned medium compared with cellular extract. Additionally, we examined expression levels of CLSTN1, CLU and NGAL of serum samples from 20 patients with lung adenocarcinoma and 10 healthy volunteers with age/sex matched. As we expected, serum levels of all three proteins were significantly higher in lung cancer patients than in healthy controls (Figure 4). The mean intensity±SD for CLSTN1, CLU and NGAL in lung cancer patients were 82.4±17.2, 110.5±23.8 and 63.5±17.7, whereas in healthy controls, the intensity was 11.3±4.1, 49.2±17.6 and 35.5±5.3, respectively. These differences were all statistically significant (*P* ≤ 0.01) with statistical power of 100%, 100% and 100%, respectively, which indicates that CLSTN1, CLU and NGAL could be potential biomarkers for lung adenocarcinoma.

### Detection of CSTN1, CLU and NGAL in tumor tissue

**Figure 5:**
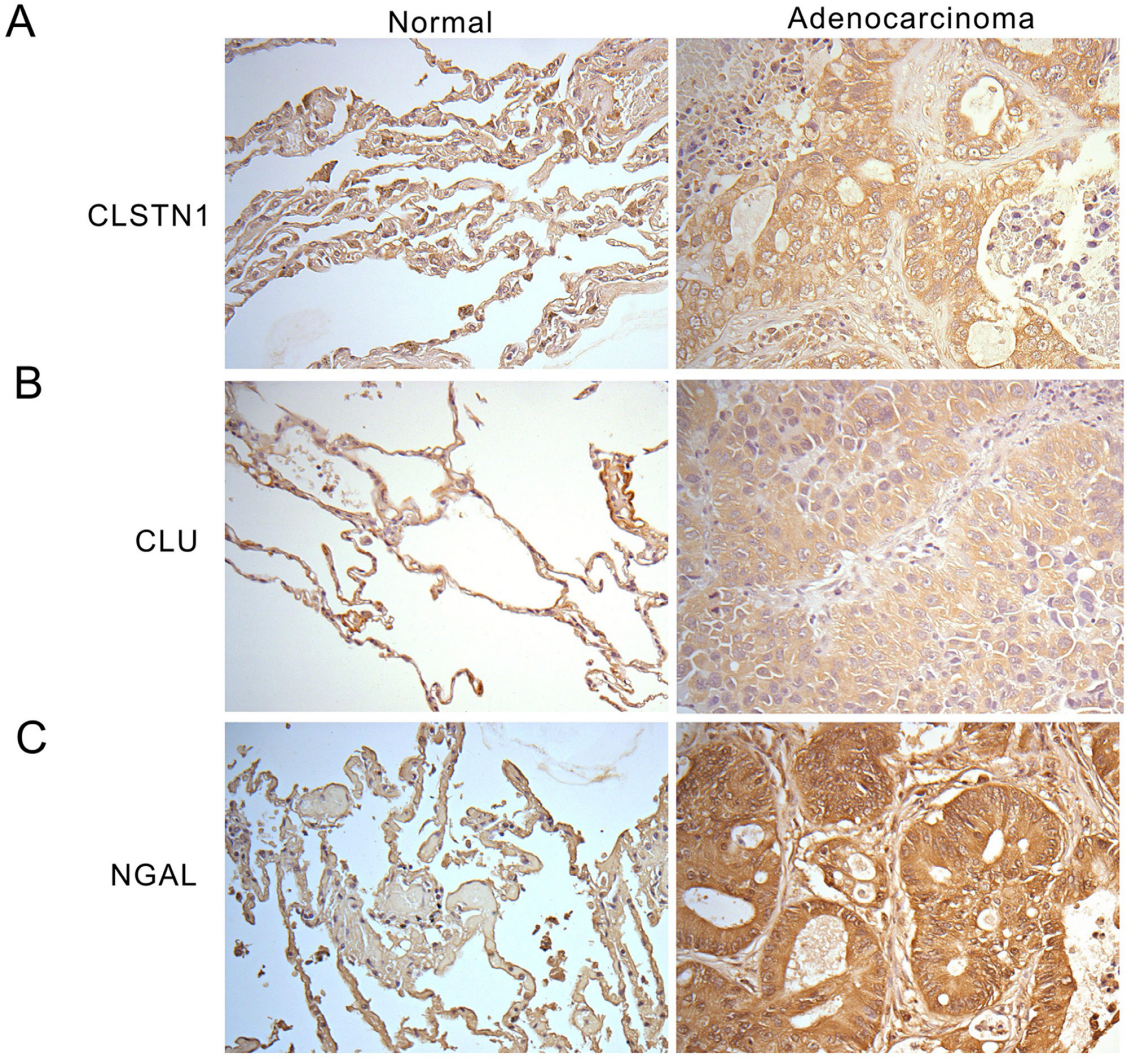
Immunohistochemistry staining of CLSTN1, CLU and NGAL in human tissue sections Tissue sections stain with **(A)** anti-CLSTN1, **(B)** anti-CLU antibodies, and **(C)** anti-NGAL antibodies. Images are showed at 200X magnification.

We next validated expression level of tumor tissues from lung cancer patients for CLSTN1, CLU and NGAL with immunohistochemistry staining. Six normal human lung tissue specimens and twelve human lung adenocarcinoma tissue specimens were used in this study. Both CLSTN1 and NGAL highly expressed in the cytoplasm of tumor cells in all (12/12) of the lung adenocarcinoma samples (Figure 5A and 5C), but not in normal alveoli cells. One the other hand, CLU was scattered expressed in the cytoplasm of tumor cells and vessel walls in all (12/12) of the lung adenocarcinoma samples although with strong signal (Figure 5B). Taken together, CLSTN1, CLU and NGAL were all highly expressed in lung cancer biopsy samples compared with normal tissues.

## DISCUSSION

In this study, we showed that tumor cells could release specific proteins into cultured medium, which may serve as potential serological biomarkers of lung adenocarcinoma. A set of 17 common proteins and 17 unique proteins with signal peptide were identified by MALDI-TOF MS proteomic assay and bioinformatics analysis from total proteins of three lung adenocarcinoma cell lines. Combined with two approaches, three proteins including CLSTN1, CLU and NGAL, were highly correlated with lung adenocarcinoma and selected for further analysis. With western blotting and immunohistochemistry staining, all three proteins in lung cancer patients significantly expressed higher than health controls. Therefore, our results suggested that CLSTN1, CLU and NGAL showed great potential to be candidate biomarkers for lung adenocarcinoma.

### Signal peptide for cancer biomarker discovery

Secretomes, a pool of protein that secreted by cells and release into culture medium, has been suggested as a permissive tool to analyzed cell real-time condition [16]. It has been reported that secreted protein may influence tumor progression through interacting with autocrine/paracrine factors and modulating the extracellular matrix composition [17]. Generally, signal peptides consist of 4-15 hydrophobic amino acid flanked by a basic N-terminus and a polar C-terminus [18]. The proteins with signal peptide, including CEA, Cyfra 21, CA 199, and CA125, were used to be targets for lung cancer diagnosis in blood screening test. Previously, bioinformatics, cell fractionation combined with microarrays or with mass spectrometry and signal sequence traps were developed into high throughput strategies to identify secreted proteins [11, 19–21]. With technique above, several secreted proteins containing signal peptide are favorable to be a potential novel prognostic biomarker for cancer [11, 12, 22, 23]. Therefore, we combined proteomic study with bioinformatics prediction of signal peptide, CLSTN1, CLU and NGAL were identified and validated with Western blot and immunohistochemistry staining. We confirmed that CLSTN1, CLU and NGAL were potential serological biomarker for lung adenocarcinoma prognostic and diagnostic.

### CLSTN1, CLU and NGAL as serological markers in lung cancer

In this study, we found CLSTN1 expression level was significantly increased in tissue and serum of 20 patients with lung adenocarcinoma compared with health control. There are three calsyntenin genes have been identified [24]. CLSTN1, calsyntenin-1, is a type-1 neuronal transmembrane protein, which is abundantly in the postsynaptic membrane [25]. CLSTN1 was originally identified as a transmitted protein that modulated synaptic signals with proteolytic cleavage fragment [26]. Although CLSTN1 was overexpressed in lung adenocarcinoma tissue and released to serum in our finding; however, the molecular mechanisms of CLSTN1 in lung cancer still need to be confirmed in the future.

CLU, known as apolipoprotein J, is a secretory glycoprotein and has been reported upregulated in various human cancers, including in breast, colon, prostate and lung [27]. Two forms of CLU are expressed in cell, which play opposite roles. The nuclear form is involved in cell apoptosis; the other (cellular/secreted) form is related to cell survival. In our study, CLU is overexpressed in cultured medium and serum samples in lung adenocarcinoma cells. Moreover, increased staining of CLU in the cytoplasm of tumor cells was detected, which indicated CUL is important for tumor cell surviving. Several studies reported CLU was expressed in endothelial cells and arterial smooth muscle cell [28, 29]. Jackson et al. demonstrated that suppression on CLU expression with antisense oligonucleotides were strongly to inhibit angiogenesis and induce apoptosis in endothelial cells [30]. Similarly, immunohistochemistry staining results also showed CLU was highly expressed in vessel cells (including endothelial cells and smooth muscle cells) in lung cancer tissues which suggested that CLU might involve in tumor angiogenesis. Previous study demonstrated that non-small cell lung cancer (NSCLC) patients showed longer lives if having higher cytoplasm clusterin expression in NSCLC tumors [31]. On the other hand, secreted clusterin has been identified as a predictor for recurrence in ovarian cancer due to its up-regulated expression in chemo-resistant tissue [32]. Furthermore, rather than cytoplasmic, secretory clusterin could promote epithelial-mesenchymal transition [33]. To sum up, these results suggested that clusterin is curial in cancer cell proliferation and angiogenesis and it also may use as a new biomarker on clinical cancer diagnostics.

Neutrophil gelatinase-associated lipocalin (NGAL), also named lipocalin-2, is a member of the lipocalin family of secreted proteins [34]. It has been reported that NGAL gene was up-regulation in various malignancies, including breast, ovarian, esophageal squamous cell carcinoma, and prostate cancer [35]. Numbers evidence showed that NGAL induced tumorigenesis through forming a disulfide-linked heterodimer with MMP-9 and destroying cellular adhesion structure [34]. Therefore, in our findings, overexpressed NGAL in both culture medium and serum samples suggest NGAL may respond to lung adenocarcinoma carcinogenesis and appeal to be anticancer target.

### Non-classical secretory pathway-exosome secretion

According to our prediction, there were 86% identified proteins without secretion leading peptides. More evidence supported that cancer-associated protein without signal peptide was able to secret from cells to matrix or medium through non-classical secretory pathway [36, 37]. Exosome is membrane vesicle from endosome that may fuse with the plasma membrane, and release the proteins into the extracellular environment in an insoluble form. The function of exosome is unclear, yet, it has been suggested playing a role in several different biological protein processes, including cancer progression [37].

### Diagram for searching candidate serologic tumor biomarkers

**Figure 6:**
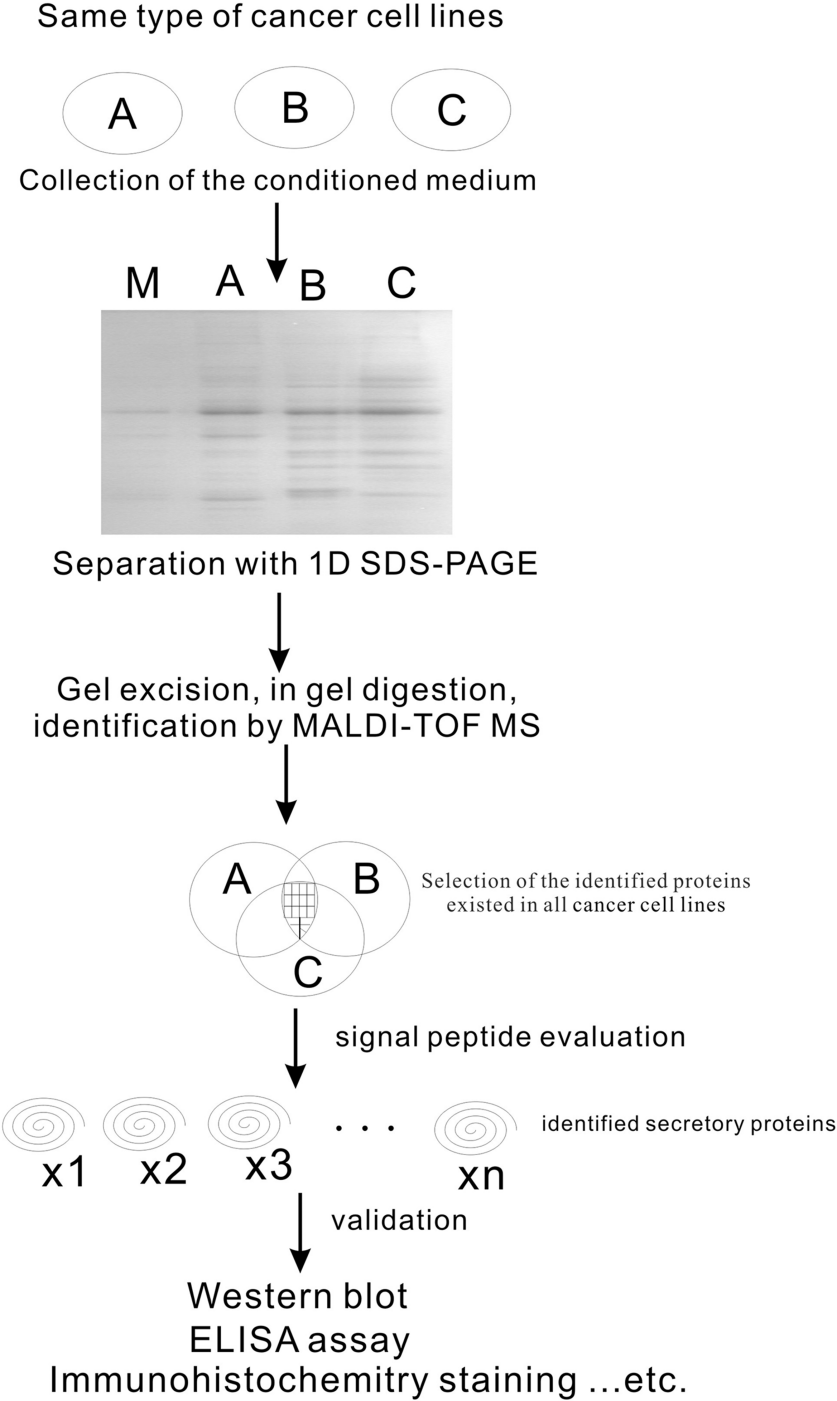
Schematic approach on identifying of serological biomarkers in lung adenocarcinoma by proteomic study

To search candidate of serological tumor biomarkers, three lung adenocarcinoma cell lines and serum samples were used in this study. Combined with proteomics and bioinformatics predication, we are successful to identify CLSTN1, CLU and NGAL as new candidate biomarkers for lung adenocarcinoma. Although, many researchers used comparison study to identify candidate biomarkers with proteomic approach on lung cancer, distinct protein profiles from cell lines and the application of proteomic study has no golden rule to follow up. We here developed a model for serological biomarker identification, which is schemed in Figure 6. Firstly, conditioned medium from cancer cell lines was collected and separated with 1D SDS-PAGE. Each sample was excised and processed with in-gel digested and MALDI-TOF MS. The common proteins existed in all cancer cell lines were chosen further prediction for signal peptides by the software of SignalP3.0. Proteins with signal peptides were then validation by Western blotting and immunohistochemistry staining with cancer samples. In conclusion, our data suggested that the higher levels of CLSTN1, CLU and NGAL in lung adenocarcinoma cultured medium as well as in serum samples tended to be tumor biomarkers for lung adenocarcinoma diagnosis.

### Limitation of this study

Thirty micrograms protein of each sample for protein identification in this study is lower than traditional 2D electrophoresis system. So, the numbers of identified protein is less than other study. However, the number (n= 34 to 49) of identified proteins on each cell line of this study shows no difference with previous report by Wu et al. [10]. In addition, we did not exclude the sample from the lymph node metastasis, where the microenvironment of tumor TNM staging, tumor infiltrated T cells, M2 macrophages and Treg may contaminate the secretions of the molecules. The patients’ underline background, *e.g.*, smoking, alcohol drinking, chronic obstructive pulmonary disease, tuberculosis and chronic metabolic inflammation could also be considered as confounding factors. The expression level of the 3 markers did not compare with other types of tumors, benign tumor, and/or various physiological/pathological conditions. Moreover, the serum subjects were comparably small in this study; additional studies with large scale may be needed to confirm our findings.

## MATERIALS AND METHODS

### Chemicals and reagents

Three lung adenocarcinoma cell lines of A549, LuCa (Lab-established cell line from a 67-year-old male with lung adenocarcinoma in right upper lobe, Chang Gung Memorial Hospital, Taiwan) and Luca Chen HCC827 were obtained from the Chang Gung Memorial Hospital, Taiwan. All analytical graded chemicals and cell culture-related reagents were from Sigma (USA). Modified trypsin of sequence grade was from Promega (USA). The syringe filter 0.45 μm was from Corning Incorporated (USA). The Bio-Rad Bradford total protein assay kit was from Bio-Rad (USA). Amicon Ultra-15 Centrifugal Filter Units were from Millipore (USA). Calsyntenin-1 (CLSTN1) was from R&D (UK), Neutrophil gelatinase-associated lipocalin (NGAL) was from Abcam (UK) and Clusterin (CLU) antibody was from Atlas (Sweden).

### Subjects

Study subjects were recruited during routine health examinations with signed consents. Thirty study subjects with different age and sex were mismatched including 20 NSCLC patients with adenocarcinoma and 10 health volunteers, enrolling for analysis. The Ethics Committee of the Taipei Tzu-Chi General Hospital approved the study.

### Cell culture condition and sample preparation

Lung adenocarcinoma cell lines were grown in RPMI 1640 medium, supplemented with 10% FBS until 70-80% confluence on 15-cm cell dishes. Cells were washed three times with 1x PBS and once with medium without serum, and incubated in the serum-free medium at 37°C for 24 hours. After incubation, the conditioned medium was carefully removed, and filtered with 0.45 μm syringe filter to remove suspended cells. A total of 45 ml conditioned medium of each cell line was then concentrated in 200 μl with Amicon Ultra-15 Centrifugal Filter Units (5000 MWCO). The protease inhibitor cocktail was immediately added into the conditioned medium and then aliquated and stored at -80°C before used. The protein concentration of the conditioned medium was measured by Bio-Rad Bradford total protein assay kit.

### One-dimensional (1D) electrophoresis

1D SDS-PAGE was performed in a 10 % gel running at 140 V for 2 hrs. Separated protein bands in the SDS-PAGE gel were visualized with coomassie blue stain.

### In gel protein digestion

The gel was excised and transferred into eppendorf. The excised gel was dried and rehydrated in 25 mM NH_4_HCO_3_ and were reduced with 50 mM dithioerythreitol at 37°C for 1 h, and then alkylated by 100 mM iodoacetamide in the dark at room temperature for one hour followed by thoroughly washing and drying. Finally, the dried gel was incubated with 10 μl of 25 mM NH_4_HCO_3_ containing 0.05 μg/μl trypsin at 37°C overnight. Peptides were extracted with 100 μl of 50% acetontitrile/5% trifluoroacetic acid. The Peptides extracts were dried in SpeedVac and resuspended with 1 μl 1% formic acid first, then add 9 μl 50% acetonitrile/0.1% formic acid for mass spectrometric analysis or stored at -80°C until used.

### Protein identification by MALDI-TOF MS

The peptide mixtures were equally mixed with a-cyano-4-hydroxycinnamic acid (Agilent Technologies Co. Ltd., USA) and spotted onto stainless steel MALDI sample target plates. Peptide mass spectra were obtained from the ABI 4800 Proteomics Analyzer MALDI-TOF/TOF MS/MS (Applied Biosystems, USA). Data acquisition, and spectral processing were carried out using Analyst and BioAnalyst™ software from Applied Biosystems.

### Database processing

Spectra were processed and analyzed using the MASCOT software (Matrix Science, London, UK) to search for the peptide mass fingerprints and MS/MS data in the Swiss port database, with the mass accuracy of within 100 ppm and as a maximum only one missed cleavage was allowed for the mass measurement. Only protein identifications with score greater than P < 0.05 were considered to be significant. Identified proteins were further analyzed to search the putative secretion signal sequences by SignalP3.0 signal peptide prediction server (http://www.cbs.dtu.dk/services/SignalP).

### Western blotting

Equal amounts of protein were loaded and separated by 10% SDS-polyacrylamide gel and transferred to polyvinylidene difluoride (PVDF) membranes. The membrane was incubated with the primary and secondary antibodies for 1 hour, respectively. The proteins were detected by ECL chemiluminescence (Amersham, USA) as described by the manufacturer. The resulting blots were quantified with Adobe Photoshop7.0 image program.

### Immunohistochemistry staining

Tissue sections removed paraffin were heated in a microwave, incubated in 2% H_2_O_2_ in ethanol, and blocked with 2% BSA, 0.1% Triton X-100, and 1% normal goat serum. The sections were incubated with CLSTN1, CLU and NGAL primary antibodies for 2 hours at 37°C (5 μg/ml) and super sensitive polymer-horseradish peroxidase (30 mines at room temperature; Biogenes, Germany), and then developed using 3, 3’-diaminobenzidine.

### Statistical analysis

The data were expressed as mean ± SD and tested by student-*t* test. p<0.05 were identified as statistically significant.

## CONCLUSION

Using the combination of the secreted proteomic study with signal peptide prediction, we provide a permissive model to find novel biomarkers of cancer. We identified that CSTN1, CLU and NGAL are candidate biomarkers for lung adenocarcinoma and may be fertile recourses of mining lung cancer research.

## SUPPLEMENTARY MATERIALS TABLES




