# Supplementary material for: Calsyntenin-1, clusterin and neutrophil gelatinase-associated lipocalin are candidate serological biomarkers for lung adenocarcinoma
Source: Oncotarget. 2017 Nov 14;8(64):107964–76. doi: 10.18632/oncotarget.22438 (PMC5746118; doi:10.18632/oncotarget.22438)
Supplement: Supplementary file 1 [file oncotarget-08-107964-s001.pdf]

Calsyntenin-1, clusterin and neutrophil gelatinase-associated lipocalin are candidate serological biomarkers for lung adenocarcinoma

SUPPLEMENTARY MATERIALS

Supplementary Table 1: Identified proteins form cultured medium of A549 cell line

See supplementary File 1

Supplementary Table 2: Identified proteins form cultured medium of Luca cell line

See supplementary File 1

Supplementary Table 3: Identified proteins form cultured medium of HCC827 cell line

See supplementary File 1
