# Supplementary material for: Calsyntenin-1, clusterin and neutrophil gelatinase-associated lipocalin are candidate serological biomarkers for lung adenocarcinoma
Source: Oncotarget. 2017 Nov 14;8(64):107964–76. doi: 10.18632/oncotarget.22438 (PMC5746118; doi:10.18632/oncotarget.22438)
Supplement: Supplementary file 2 [file oncotarget-08-107964-s002.docx]

**Supplementary data**

Table S1. Identified proteins form cultured medium of A549 cell line.

| Protein name | Score | P value | Cover rate(%) | PI | MW  (Da) |
| --- | --- | --- | --- | --- | --- |
| (O15230)Laminin alpha-5 chain | 65 | 0.004 | 4 | 6.59 | 412214 |
| (O94985) Calsyntenin-1 precursor | 104 | 4.80E-07 | 14 | 4.81 | 111000 |
| (O95613) Pericentrin 2 | 60 | 0.014 | 9 | 5.39 | 380692 |
| alpha-actinin L | 135 | 3.80E-10 | 22 | 5.27 | 105253 |
| (Q08380) Galectin-3 binding protein precursor | 54 | 0.05 | 12 | 5.13 | 66217 |
| (P07900) Heat shock protein HSP 90-alpha (HSP 86) | 59 | 0.015 | 7 | 4.94 | 84896 |
| (P08238) Heat shock protein HSP 90-beta (HSP 84) | 52 | 0.036 | 5 | 4.97 | 83429 |
| (P26038) Moesin | 70 | 0.0012 | 17 | 6.09 | 67763 |
| (P15311) Ezrin (p81) (Cytovillin) (Villin 2) | 69 | 0.0014 | 17 | 5.95 | 69341 |
| (Q8IZT6) Abnormal spindle | 64 | 0.0051 | 11 | 10.45 | 413255 |
| (P35241) Radixin | 58 | 0.019 | 12 | 6.03 | 68637 |
| (P29401) Transketolase | 77 | 0.00023 | 15 | 7.58 | 68531 |
| (P14618) Pyruvate kinase, isozymes M1/M2 | 148 | 1.90E-11 | 19 | 7.95 | 58349 |
| (P00352) Retinal dehydrogenase 1 | 125 | 3.80E-09 | 15 | 6.29 | 55334 |
| (P06733) Alpha enolase | 81 | 9.80E-05 | 14 | 6.99 | 47356 |
| (p60709) actin beta | 313 | 6.00E-28 | 23 | 5.29 | 42058 |
| (P63261) Actin, cytoplasmic 2 (Gamma-actin) | 313 | 6.00E-28 | 23 | 5.31 | 42114 |
| (P68032) Actin, alpha cardiac (Alpha-cardiac actin) | 196 | 3.00E-16 | 18 | 5.23 | 42340 |
| (P68133) Actin, alpha skeletal muscle (Alpha-actin 1) | 196 | 3.00E-16 | 18 | 5.23 | 42372 |
| SM GAMMA-ACTIN | 173 | 6.00E-14 | 9 | 5.31 | 42256 |
| (P62736) Actin, aortic smooth muscle (Alpha-actin 2) | 173 | 6.00E-14 | 9 | 5.23 | 42388 |
| (P10909) Clusterin precursor | 277 | 2.40E-24 | 31 | 5.89 | 53041 |
| (P04075) Fructose-bisphosphate aldolase A | 59 | 1.40E-02 | 19 | 8.39 | 39728 |
| (O60218) Aldo-keto reductase family 1 member B10 | 154 | 4.80E-12 | 26 | 7.12 | 36230 |
| (P04406) Glyceraldehyde-3-phosphate dehydrogenase, liver | 57 | 2.60E-02 | 23 | 8.58 | 36073 |
| (P07355) Annexin A2 | 126 | 3.00E-09 | 39 | 7.56 | 38681 |
| (P63104) 14-3-3 protein zeta/delta | 90 | 1.20E-05 | 23 | 4.73 | 27902 |
| (P61981) 14-3-3 protein zeta/delta | 96 | 3.20E-06 | 23 | 4.8 | 28328 |
| (P60174) Triosephosphate isomerase | 120 | 1.20E-08 | 31 | 6.51 | 26812 |
| (Q06830) Peroxiredoxin 1 | 97 | 2.50E-06 | 30 | 8.27 | 22328 |
| (P09211) Glutathione S-transferase P | 63 | 6.20E-03 | 22 | 5.44 | 23442 |
| (P22392) Nucleoside diphosphate kinase B | 65 | 3.50E-03 | 25 | 8.52 | 17403 |
| (P15531) Nucleoside diphosphate kinase A | 60 | 1.30E-02 | 19 | 5.83 | 17312 |

Table S2. Identified proteins form cultured medium of Luca cell line.

| Protein name | Score | P value | Cover rate (%) | PI | MW  (Da) |
| --- | --- | --- | --- | --- | --- |
| (P07996) Thrombospondin-1 | 109 | 1.50E-07 | 10 | 4.71 | 133390 |
| (O94985) Calsyntenin-1 | 60 | 0.014 | 8 | 4.81 | 111000 |
| (P13639) Elongation factor 2 (EF-2) | 86 | 3.00E-05 | 13 | 6.42 | 96132 |
| (P07900) Heat shock protein HSP 90-alpha (HSP 86) | 362 | 7.60E-33 | 40 | 4.94 | 84896 |
| (P08238) Heat shock protein HSP 90-beta | 371 | 9.60E-34 | 31 | 4.97 | 83429 |
| (Q12931) Heat shock protein 75 kDa, | 60 | 1.10E-02 | 11 |  | 80251 |
| (Q00325) Phosphate carrier protein (PTP) | 55 | 4.00E-02 | 21 | 9.45 | 40533 |
| (Q9GZU8) NEFA-interacting nuclear protein (NIP 30) | 57 | 2.30E-02 | 26 | 5.38 | 29127 |
| (P11142) Heat shock cognate 71 kDa protein | 124 | 4.80E-09 | 26 | 5.37 | 71086 |
| (P54652) Heat shock-related 70 kDa protein 2 | 57 | 2.20E-02 | 13 | 5.56 | 70268 |
| (P48741) Heat shock 70 kDa protein 7 | 76 | 3.10E-04 | 23 | 6.97 | 27006 |
| (P17066) Heat shock 70 kDa protein 6 | 85 | 3.90E-05 | 10 | 5.81 | 71448 |
| (P29401) Transketolase | 63 | 6.60E-03 | 16 | 7.58 | 68531 |
| (P34931) Heat shock 70 kDa protein 1L | 65 | 4.20E-03 | 12 | 5.76 | 70737 |
| (Q8NBP7) Proprotein convertase subtilisin/kexin type 9 precursor | 123 | 6.00E-09 | 17 | 6.09 | 75833 |
| (Q8WX94) NACHT-, LRR- and PYD-containing protein 7 | 58 | 2.00E-02 | 15 | 5.87 | 113939 |
| (P14618) Pyruvate kinase, isozymes M1/M2 | 247 | 2.40E-21 | 44 | 7.95 | 58349 |
| (P11413) Glucose-6-phosphate 1-dehydrogenase | 167 | 2.40E-31 | 38 | 6.44 | 59561 |
| (P30838) Aldehyde dehydrogenase, dimeric NADP-preferring | 94 | 4.60E-66 | 20 | 6.11 | 50753 |
| (Q9BQE3) Tubulin alpha-6 chain | 68 | 1.90E-03 | 17 | 4.96 | 50560 |
| (P68363) Tubulin alpha-ubiquitous chain | 74 | 4.80E-04 | 19 | 4.94 | 50816 |
| (Q71U36) Tubulin alpha-3 chain | 68 | 1.90E-03 | 17 | 4.94 | 50800 |
| (P07437) Tubulin beta-2 chain | 174 | 4.80E-14 | 34 | 4.78 | 50103 |
| (P68371) Tubulin beta-4b chain | 161 | 9.60E-13 | 31 | 4.79 | 50263 |
| (Q13509) Tubulin beta-3 chain | 107 | 2.40E-07 | 24 | 4.83 | 50864 |
| (P04350) Tubulin beta-4 chain | 97 | 2.30E-06 | 22 | 4.81 | 50063 |
| (P06733) Alpha enolase | 462 | 7.60E-43 | 40 | 6.99 | 47356 |
| (Q05524) Alpha enolase, lung specific | 98 | 2.00E-06 | 16 | 5.78 | 49852 |
| (P13929) Beta enolase | 62 | 8.10E-03 | 19 | 7.73 | 47174 |
| (P52209) 6-phosphogluconate dehydrogenase, decarboxylating | 139 | 1.50E-10 | 31 | 6.88 | 53497 |
| (P63261) Actin, cytoplasmic 2 | 348 | 1.90E-31 | 50 | 5.31 | 42114 |
| (P68032) Actin, alpha cardiac | 276 | 3.00E-24 | 42 | 5.23 | 42340 |
| (P68133) Actin, alpha skeletal muscle | 264 | 4.80E-23 | 36 | 5.23 | 42372 |
| (P63267) Actin, gamma-enteric smooth muscle | 348 | 1.90E-31 | 50 | 5.31 | 42256 |
| (P62736) Actin, aortic smooth muscle | 225 | 3.80E-19 | 39 | 5.23 | 42388 |
| (P04075) Fructose-bisphosphate aldolase A | 242 | 7.60E-21 | 35 | 8.39 | 39728 |
| (P09972) Fructose-bisphosphate aldolase C | 58 | 2.10E-02 | 15 | 6.46 | 39706 |
| (P42330) Aldo-keto reductase family 1 member C3 | 291 | 9.60E-26 | 56 | 8.05 | 37227 |
| (Q04828) Aldo-keto reductase family 1 member C1 | 247 | 2.40E-21 | 43 | 8.02 | 37229 |
| (P52895) Aldo-keto reductase family 1 member C2 | 79 | 1.50E-04 | 37 | 7.13 | 37118 |
| (P17516) Aldo-keto reductase family 1 member C4 | 66 | 2.70E-03 | 11 | 6.17 | 37477 |
| (P04406) Glyceraldehyde-3-phosphate dehydrogenase, liver | 113 | 6.00E-08 | 34 | 8.58 | 36073 |
| (O60218) Aldo-keto reductase family 1 member B10 | 65 | 3.80E-03 | 21 | 7.12 | 36230 |
| (P40925) Malate dehydrogenase, cytoplasmic | 56 | 2.90E-02 | 14 | 6.89 | 36504 |
| (P04792) Heat-shock protein beta-1 (HspB1) (Heat shock 27 kDa protein) | 91 | 1.00E-05 | 40 | 5.98 | 22826 |
| (P63104) 14-3-3 protein zeta/delta | 101 | 9.60E-07 | 28 | 4.73 | 27902 |
| (Q9UPN3) Microtubule-actin crosslinking factor 1, isoforms 1/2/3/5 | 66 | 3.10E-03 | 6 | 5.27 | 623688 |
| (Q96PK2) Microtubule-actin crosslinking factor 1, isoforms 4 | 54 | 5.00E-02 | 8 | 5.2 | 673796 |
| (P60174) Triosephosphate isomerase | 100 | 1.20E-06 | 46 | 6.51 | 26812 |
| (Q06830) Peroxiredoxin 1 | 269 | 1.50E-23 | 44 | 8.27 | 22328 |
| (P09211) Glutathione S-transferase P | 217 | 2.40E-18 | 44 | 5.44 | 23442 |
| (Q13162) Peroxiredoxin 4 | 88 | 2.00E-05 | 12 | 5.86 | 30753 |
| Pericentrin 2 | 66 | 3.20E-03 | 10 | 5.39 | 380692 |
| (P07996) Thrombospondin-1 precursor | 109 | 1.50E-07 | 10 | 4.71 | 133390 |
| (O94985) Calsyntenin-1 precursor | 60 | 0.014 | 8 | 4.81 | 111000 |
| (P13639) Elongation factor 2 (EF-2) | 86 | 3.00E-05 | 13 | 6.42 | 96132 |
| (P07900) Heat shock protein HSP 90-alpha (HSP 86) | 362 | 7.60E-33 | 40 | 4.94 | 84896 |
| (P08238) Heat shock protein HSP 90-beta | 371 | 9.60E-34 | 31 | 4.97 | 83429 |
| (Q12931) Heat shock protein 75 kDa, | 60 | 1.10E-02 | 11 | 4.93 | 80251 |
| (Q00325) Phosphate carrier protein (PTP) | 55 | 4.00E-02 | 21 | 9.45 | 40533 |
| (Q9GZU8) NEFA-interacting nuclear protein (NIP 30) | 57 | 2.30E-02 | 26 | 5.38 | 29127 |
| (P11142) Heat shock cognate 71 kDa protein | 124 | 4.80E-09 | 26 | 5.37 | 71086 |
| (P54652) Heat shock-related 70 kDa protein 2 | 57 | 2.20E-02 | 13 | 5.56 | 70268 |
| (P48741) Heat shock 70 kDa protein 7 | 76 | 3.10E-04 | 23 | 6.97 | 27006 |
| (P17066) Heat shock 70 kDa protein 6 | 85 | 3.90E-05 | 10 | 5.81 | 71448 |

Table S3. Identified proteins form cultured medium of HCC827 cell line

| Protein name | Score | P value | Cover  rate (%) | | | PI | | MW  (Da) | |  |
| --- | --- | --- | --- | --- | --- | --- | --- | --- | --- | --- |
| (O00468) Agrin precursor | 85 | 4.00E-05 | | 11 | | 6.05 | | 223044 | |  |
| (Q14549) Homeobox protein GBX-1 | 55 | 0.036 | | | 59 | | 11.02 | | 15387 | |
| (P24821) Tenascin precursor | 142 | 7.60E-11 | | 15 | | 4.79 | | 246456 | |  |
| (Q9UPN3) Microtubule-actin crosslinking factor 1, isoforms 1/2/3/5 | 60 | 0.012 | | 8 | | 5.27 | | 623688 | |  |
| (O75665) Oral-facial-digital syndrome 1 protein | 76 | 2.70E-04 | | 24 | | 5.82 | | 117063 | |  |
| (P53396) ATP-citrate synthase | 63 | 0.0056 | | 17 | | 6.95 | | 121676 | |  |
| (P19338) Nucleolin | 91 | 8.70E-06 | | 14 | | 4.59 | | 76225 | |  |
| (O94985) Calsyntenin-1 precursor | 81 | 9.80E-05 | | 15 | | 4.18 | | 111000 | |  |
| (P08238) Heat shock protein HSP 90-beta | 202 | 7.60E-17 | | 25 | | 4.97 | | 83429 | |  |
| (P07900) Heat shock protein HSP 90-alpha (HSP 86) | 184 | 4.80E-15 | | 21 | | 4.94 | | 84896 | |  |
| (P15586) N-acetylglucosamine-6-sulfatase precurso | 148 | 1.90E-11 | | 13 | | 8.6 | | 62854 | |  |
| (P13639) Elongation factor 2 (EF-2) | 74 | 5.30E-04 | | 16 | | 6.42 | | 96132 | |  |
| (Q12931) Heat shock protein 75 kDa, mitochondrial precursor | 66 | 2.80E-03 | | 13 | | 8.05 | | 80251 | |  |
| (P11021) 78 kDa glucose-regulated protein precursor | 236 | 3.00E-20 | | 28 | | 5.07 | | 72404 | |  |
| (P14618) Pyruvate kinase, isozymes M1/M2 | 132 | 7.60E-10 | | 29 | | 7.95 | | 58349 | |  |
| (P19878) Neutrophil cytosol factor 2 (NCF-2) | 69 | 1.60E-03 | | 27 | | 5.88 | | 60246 | |  |
| (P20929) Nebulin | 54 | 4.60E-02 | | 8 | | 9.1 | | 775802 | |  |
| (P07437) Tubulin beta-2 chain | 197 | 2.40E-16 | | 37 | | 4.78 | | 50103 | |  |
| (P68371) Tubulin beta-? chain | 196 | 3.00E-16 | | 36 | | 4.79 | | 50263 | |  |
| (Q9BQE3) Tubulin alpha-6 chain | 148 | 1.90E-11 | | 31 | | 4.96 | | 50560 | |  |
| (P68363) Tubulin alpha-ubiquitous chain | 139 | 1.50E-10 | | 26 | | 4.94 | | 50816 | |  |
| (Q71U36) Tubulin alpha-3 chain | 132 | 7.6e-0410 | | 23 | | 4.94 | | 50800 | |  |
| (Q13509) Tubulin beta-3 chain | 116 | 3.00E-08 | | 23 | | 4.83 | | 50864 | |  |
| (P04350) Tubulin beta-4 chain | 84 | 5.00E-05 | | 28 | | 4.81 | | 50063 | |  |
| (Q9UPN3) Microtubule-actin crosslinking factor 1, isoforms 1/2/3/5 | 68 | 0.0017 | | 9 | | 5.27 | | 623688 | |  |
| (P06733) Alpha enolase | 396 | 3.00E-36 | | 41 | | 6.99 | | 47356 | |  |
| (Q05524) Alpha enolase, lung specific | 93 | 6.50E-06 | | 20 | | 5.78 | | 49852 | |  |
| (P13929) Beta enolase | 74 | 0.0049 | | 21 | | 7.73 | | 47174 | |  |
| Actin Gamma | 421 | 9.60E-39 | | 53 | | 5.31 | | 42256 | |  |
| (P63261) Actin, cytoplasmic 2 | 409 | 1.50E-37 | | 48 | | 5.31 | | 42114 | |  |
| (P68032) Actin, alpha cardiac | 232 | 7.60E-20 | | 32 | | 5.23 | | 42340 | |  |
| (P68133) Actin, alpha skeletal muscle | 231 | 9.60E-20 | | 32 | | 5.23 | | 42372 | |  |
| (P62736) Actin, aortic smooth muscle | 175 | 3.80E-14 | | 29 | | 5.23 | | 42388 | |  |
| Actin smooth muscle | 175 | 3.80E-14 | | 30 | |  | |  | |  |
| (P00749) Urokinase-type plasminogen activator precursor | 62 | 0.0073 | | 19 | | 8.78 | | 49944 | |  |
| (P00558) Phosphoglycerate kinase 1 | 79 | 0.0016 | | 18 | | 8.3 | | 44861 | |  |
| (P07205) Phosphoglycerate kinase 2 | 79 | 1.70E-04 | | 11 | | 8.74 | | 45042 | |  |
| (O94833) Bullous pemphigoid antigen 1, isoforms 6/9/10 | 71 | 0.00093 | | 9 | | 5.5 | | 593719 | |  |
| (P04075) Fructose-bisphosphate aldolase A | 182 | 7.60E-15 | | 40 | | 8.39 | | 39728 | |  |
| (P09972) Fructose-bisphosphate aldolase C | 56 | 0.032 | | 35 | | 6.46 | | 39706 | |  |
| (P04083) Annexin A1 | 248 | 1.90E-21 | | 42 | | 6.64 | | 38791 | |  |
| (P04406) Glyceraldehyde-3-phosphate dehydrogenase, liver | 164 | 4.80E-13 | | 41 | | 8.58 | | 36073 | |  |
| (P05534) HLA class I histocompatibility antigen | 54 | 4.90E-02 | | 19 | | 5.91 | | 40953 | |  |
| Annexin A2 | 55 | 3.50E-02 | | 31 | | 7.56 | | 38681 | |  |
| (P12429) Annexin A3 | 82 | 8.30E-05 | | 32 | | 5.63 | | 36396 | |  |
| (Q9UPN3) Microtubule-actin crosslinking factor 1, isoforms 1/2/3/5 | 107 | 2.40E-07 | | 10 | | 5.27 | | 623688 | |  |
| (P04792) Heat-shock protein beta-1 | 68 | 1.90E-03 | | 40 | | 5.98 | | 22826 | |  |
| (Q96PK2) Microtubule-actin crosslinking factor 1, isoform 4 | 63 | 6.20E-03 | | 5 | | 5.2 | | 673796 | |  |
| (Q8WXH0) Nesprin 2 | 58 | 2.00E-02 | | 8 | | 5.26 | | 801860 | |  |
| (P09211) Glutathione S-transferase P | 99 | 1.70E-06 | | 38 | | 5.44 | | 23442 | |  |
| (P80188) Neutrophil gelatinase-associated lipocalin precursor (NGAL) | 342 | 7.60E-31 | | 42 | | 9.02 | | 22748 | |  |
| (P60174) Triosephosphate isomerase | 65 | 4.00E-03 | | 36 | | 6.51 | | 26812 | |  |
